# Supplementary material for: Homotypic and heterotypic immune responses to Omicron variant in immunocompromised patients in diverse clinical settings
Source: Nat Commun. 2022 Aug 4;13:4489. doi: 10.1038/s41467-022-32235-x (PMC9352686; doi:10.1038/s41467-022-32235-x)

SUPPLEMENTARY INFORMATION

Supplementary Table 1 - Comparison of baseline characteristics of transplant patient cohorts. IQR: Interquartile range, SD: Standard deviation. Cohort 1 includes transplant recipients with COVID-19 before the Omicron wave; Cohort 2 includes transplant recipients with BA.1 infection; Cohort 3 includes uninfected transplant recipients with 3 doses of mRNA-1273 (Moderna) vaccine; ƚ Years from transplant to infection in case of cohort 1 and 2, and from transplant to last dose of the vaccine in case of cohort 3. ¥ Continuous variables p-value estimated using two-sided Mann Whitney U-test (Wilcoxon rank-sum). Categorical variables p-value estimated using two-sided Fisher's exact test.

Characteristics	Cohort 1 (N=91)	Cohort 2 (N=75)	Cohort 3 (N=60)	p value¥
Age – years (mean±SD)	54.2±14.1	53.5±12.7	67.7±4.8	<0.001
Female sex – no. (%)	23 (25%)	26 (35%)	23 (38%)	0.19
Type of transplant – no. (%)				
Kidney or KP	52 (57.1%)	39 (52%)	35 (58.3%)	0.72
Lung	16 (17.6%)	16 (21.3%)	11 (18.3%)	0.83
Liver	19 (20.9%)	11 (14.7%)	3 (5%)	0.019
Heart	3 (3.3%)	6 (8%)	10 (16.7%)	0.017
Kidney-liver	1 (1.1%)	3 (4%)	1 (1.67%)	0.53
Years since transplant (median, IQR)ƚ	5.4 (2.3-9.1)	5.9 (2.3-9.9)	3.8 (2.0-6.7)	0.29
Immunosuppressant – no. (%)				
Prednisone	73 (80.2%)	64 (85.3%)	50 (83.3%)	0.68
Calcineurin-inhibitor (Tacrolimus or cyclosporine)	84 (92.3%)	73 (97.3%)	59 (98.3%)	0.17
Anti-metabolite (Mycophenolate or azathioprine)	74 (81.3%)	64 (85.3%)	51 (85%)	0.79
Antilymphocyte globulin in the previous 3 months	4 (4.4%)	1 (1.3%)	0 (0%)	0.22
Acute rejection in the previous 3 months	2 (2.2%)	0	1(1.7%)	0.49
B-cell depleting antibody in the previous 3 months (rituximab)	0	0	0	1.00

Supplementary Table 2 – Comparison of baseline characteristics of SOTR with and without T-cell testing in Cohort 1. IQR: Interquartile range. SD: Standard deviation. SOTR:solid organ transplant recipients. ł Years from transplant to infection. ¥ Continuous variables p-value estimated using a two-sided Mann Whitney U-test (Wilcoxon rank-sum). Categorical variables p-value estimated using two-sided Fisher's exact test.

Characteristics (Cohort 1)	T-cell analysis (N=25)	No T-cell analysis (N=66)	p value¥
Age – years (mean±SD)	55 ± 14.1	53.8 ± 14.2	0.72
Female sex – no. (%)	7 (28%)	16 (24%)	0.79
Type of transplant – no. (%)			
Kidney or KP	15 (60%)	37 (56%)	0.81
Lung	5 (20%)	11 (17%)	0.76
Liver	5 (20%)	14 (21%)	1.00
Heart	0 (0%)	3 (5%)	0.56
Kidney-liver	0 (0%)	1 (2%)	1.00
Years since transplant (median, IQR)ł	3.7 (1.8-6.6)	5.7 (2.9-9.4)	0.14
Immunosuppressant – no. (%)			
Prednisone	20 (80%)	53 (80%)	1.00
Calcineurin-inhibitor (Tacrolimus or cyclosporine)	24 (96%)	60 (91%)	0.67
Anti-metabolite (Mycophenolate or azathioprine)	22 (33%)	52 (79%)	0.38
Sirolimus	0 (0%)	1 (2%)	1.00
Antilymphocyte globulin in the previous 3 months	0 (0%)	4 (6%)	0.57
Acute rejection in the previous 3 months	0 (0%)	2 (3%)	1.00
B-cell depleting antibody in the previous 3 months (rituximab)	0 (0%)	0 (0%)	1.00

Supplementary Table 3 - Polyfunctional $CD4^+$ T-cell responses by baseline characteristics in Cohort 2. Proportions of low (<100 per 10^6), medium (100-1000 per 10^6), and high (>1000 per 10^6) BA.1-specific polyfunctional $CD4^+$ T-cell frequencies are shown by baseline demographics. Continuous variables p-value estimated using two-sided Mann Whitney U-test (Wilcoxon rank-sum). Categorical variables p-value estimated using two-sided Fisher's exact test.

**Characteristics**	**Low (N=5)**	**Medium (N=32)**	**High (N=27)**	**P-value**
Age – years (mean±SD)	44.9±11.6	53.8±12.0	52.9±12.9	0.34
Female sex – n (%)	4(80%)	9 (28%)	8 (30%)	0.082
Type of transplant – n (%)				
Kidney	1 (20%)	12 (38%)	12 (44%)	0.64
Lung	3 (60%)	6 (19%)	4 (15%)	0.088
Liver	0 (0%)	4 (13%)	6 (22%)	0.49
Heart	1(20%)	2 (6%)	2 (7%)	0.45
Kidney-pancreas	0 (0%)	6 (19%)	3 (11%)	0.54
Kidney-liver	0 (0%)	2(6%)	0 (0%)	0.57
Years since transplant (mean (SD))	6.2 (2.9)	8.9 (9.0)	7.1 (7.8)	0.62
Immunosuppressant – n (%)				
Prednisone	5 (100%)	28 (88%)	22 (81%)	0.65
Calcineurin Inhibitor	5 (100%)	31(97%)	27 (100%)	1.00
Anti-metabolite	3 (60%)	27 (84%)	24 (89%)	0.22
Anti-lymphocyte globulin in the last 3 months	0 (0%)	0 (0%)	1 (4%)	0.50
No. of COVID-19 vaccines – n (%)				
0	0 (0%)	0 (0%)	2 (7%)	0.69
1	0 (0%)	0 (0%)	1 (4%)	
2	1 (20%	4 (13%)	5 (19%)	
3	4 (80%)	25 (78%)	17 (63%)	
4	0 (0%)	3 (9%)	2 (7%)	

Supplementary Table 4 - Polyfunctional CD8$^+$ T-cell responses by baseline characteristics in Cohort 2. Negative denotes <100 per 10^6 positive cells, and positive denotes ≥100 per 10^6 positive cells. Continuous variables p-value estimated using two-sided Mann Whitney U-test (Wilcoxon rank-sum). Categorical variables p-value estimated using two-sided Fisher's exact test.

	**Negative (N=49)**	**Positive (N=15)**	**P-value**
Age – years (mean±SD)	52.3±13.0	53.8±10.0	0.69
Female sex – no. (%)	17(35%)	4 (27%)	
Type of Transplant n (%)			
Kidney	18 (37%)	7 (47%)	0.55
Lung	9 (18%)	4 (27%)	0.48
Liver	6 (12%)	4 (27%)	0.23
Heart	5 (10%)	0 (0%)	0.33
Kidney-pancreas	9 (18%)	0 (0%)	0.10
Kidney-liver	2 (4%)	0 (0%)	1.00
Years since transplant (mean (SD))	8.4 (8.5)	6.4 (6.9)	0.41
Immunosuppressant n (%)			
Prednisone	42 (86%)	13 (87%)	1.00
Calcineurin Inhibitor	48(98%)	15 (100%)	1.00
Anti-metabolite	40 (82%)	14 (93%)	0.43
Anti-lymphocyte globulin in the last 3 months	1 (2%)	0 (0%)	1.00
Number of vaccine doses n (%)			0.79
0	2 (4%)	0 (0%)	
1	1 (2%)	0 (0%)	
2	7 (14%)	3 (20%)	
3	36 (73%)	10 (67%)	
4	3 (6%)	2 (13%)	

Supplementary Figure 1 – Figure exemplifying the gating strategy for detecting intracellular cytokines. Arrows indicate the sequence of hierarchical gates. Number in each plot refers to the frequency of the gated subset. CD4$^+$ and CD8$^+$ T-cell plots show T-cells stimulated with wild-type or Omicron BA.1 SARS-CoV-2 spike peptides, or negative control.

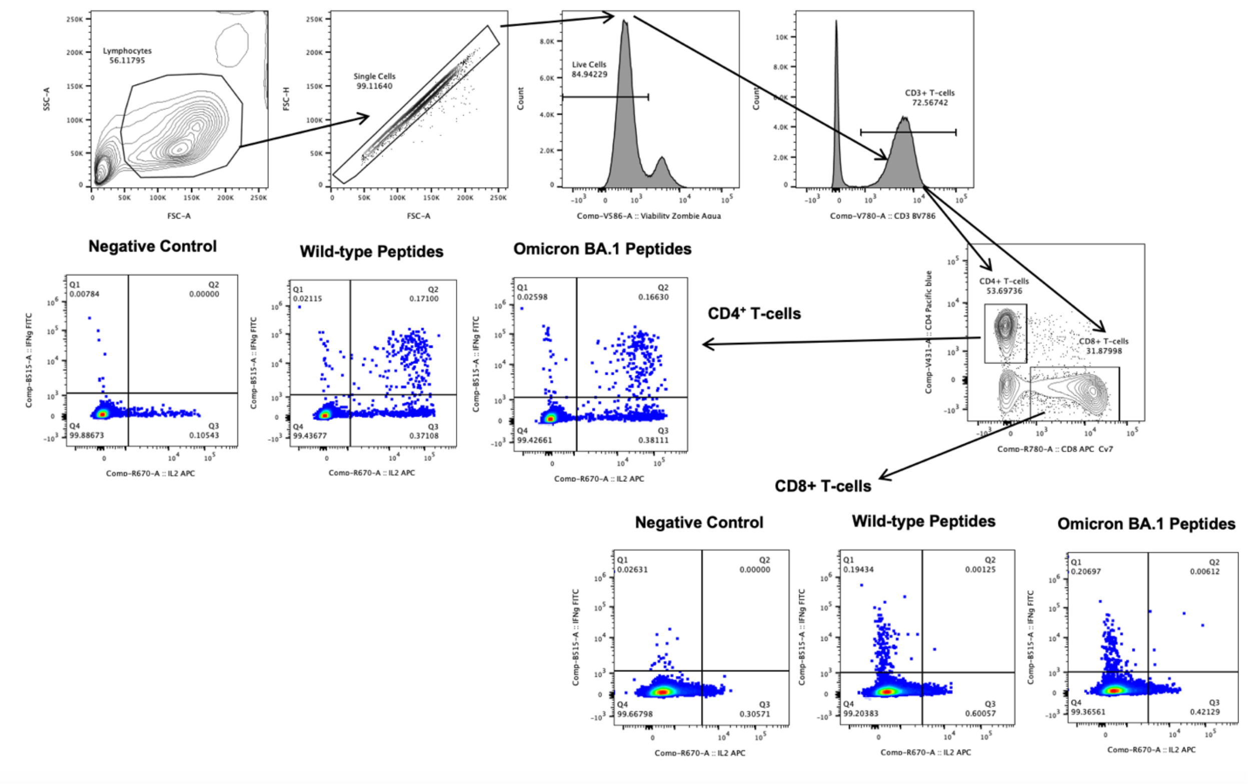

Supplementary Figure 2 – Anti-nucleocapsid antibody levels in Cohort 3, transplant recipients receiving three doses of mRNA vaccine with no prior history of SARS-CoV-2 infection. Dashed horizontal line shows the threshold for test positivity, according to manufacturer's instructions (≥1.4 U/mL). Each dot represents one patient, n=60. Source data are provided in the Source Data file.

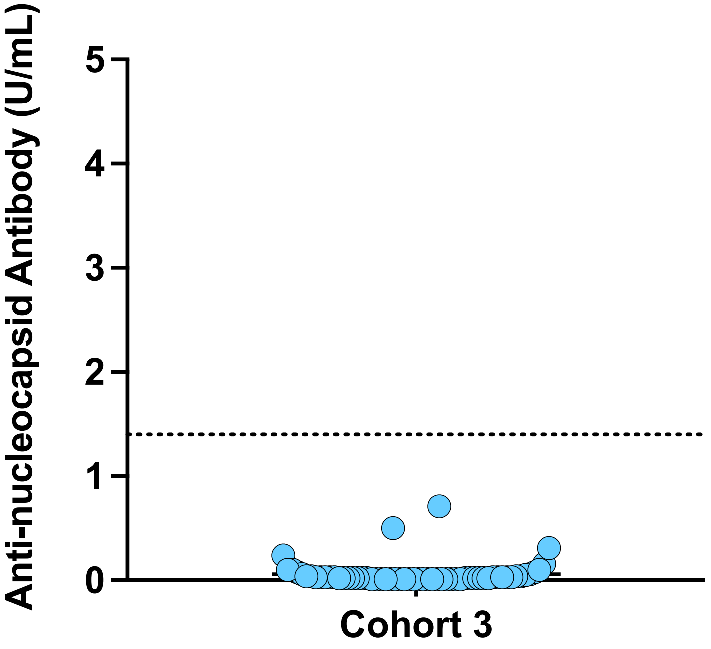

Supplementary Figure 3 – Two-sided Spearman correlation of BA.1 neutralizing antibody titers (log_{10} ID50) with SARS-CoV-2 spike-specific polyfunctional $CD4^+$ (A) and $CD8^+$ (B) T-cells frequencies. Source data are provided in the Source Data file.

A

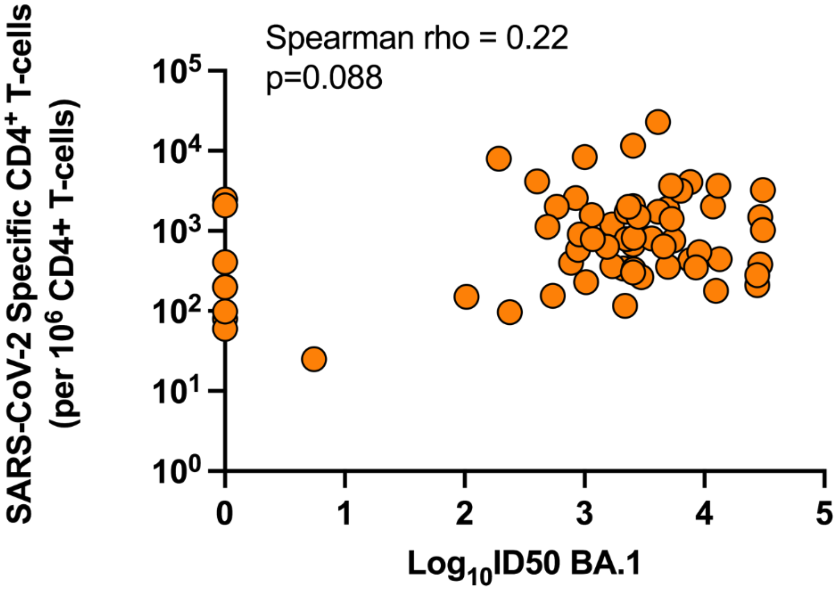

B

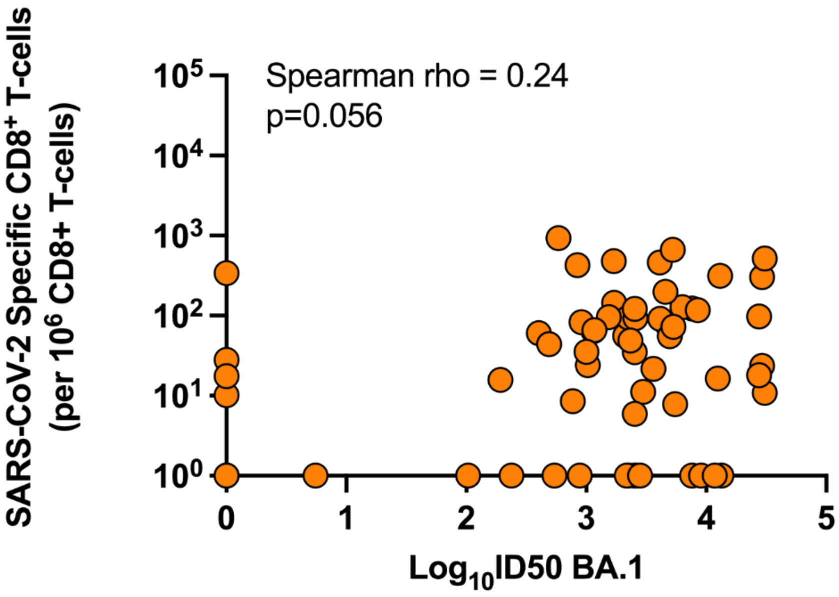

Supplementary Figure 4 – Correlation of BA.1 and BA.2 neutralizing antibody levels in Cohorts 3 and 4. (A) Correlation between the log_{10}ID50 of neutralizing antibody titers against Omicron BA.1 (x-axis) and BA.2 (y-axis) in transplant patients who are triple vaccinated and uninfected (Cohort 3; n=60). Diagonal line denotes line of best-fit by regression; (B) in triple vaccinated immunocompetent health care workers (Cohort 4; n=20). All correlations were performed using a two-sided Spearman test, p<0.001 for both correlations shown. Source data are provided in the Source Data file.

A

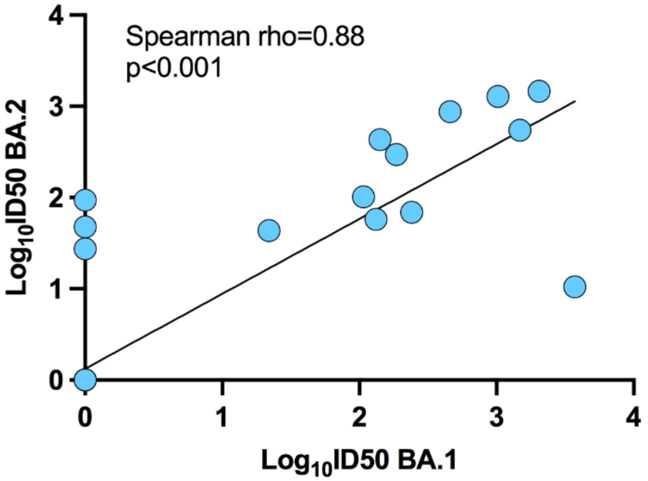

B

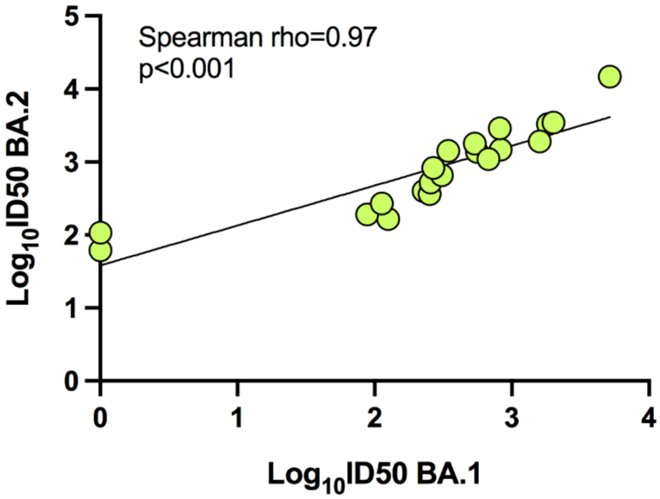

Supplement: Supplementary file 1 — Supplementary Information [file 41467_2022_32235_MOESM1_ESM.pdf]
