# Supplementary material for: Homotypic and heterotypic immune responses to Omicron variant in immunocompromised patients in diverse clinical settings
Source: Nat Commun. 2022 Aug 4;13:4489. doi: 10.1038/s41467-022-32235-x (PMC9352686; doi:10.1038/s41467-022-32235-x)
Supplement: Supplementary file 3 — Reporting Summary [file 41467_2022_32235_MOESM3_ESM.pdf]

nature portfolio

Corresponding author(s): Atul Humar
Last updated by author(s): June 21, 2022

Reporting Summary

Nature Portfolio wishes to improve the reproducibility of the work that we publish. This form provides structure for consistency and transparency in reporting. For further information on Nature Portfolio policies, see our Editorial Policies and the Editorial Policy Checklist.

Statistics

For all statistical analyses, confirm that the following items are present in the figure legend, table legend, main text, or Methods section.

n/a	Confirmed	
☐	☒	The exact sample size (*n*) for each experimental group/condition, given as a discrete number and unit of measurement
☐	☒	A statement on whether measurements were taken from distinct samples or whether the same sample was measured repeatedly
☐	☒	The statistical test(s) used AND whether they are one- or two-sided<br>*Only common tests should be described solely by name; describe more complex techniques in the Methods section.*
☐	☒	A description of all covariates tested
☐	☒	A description of any assumptions or corrections, such as tests of normality and adjustment for multiple comparisons
☐	☒	A full description of the statistical parameters including central tendency (e.g. means) or other basic estimates (e.g. regression coefficient) AND variation (e.g. standard deviation) or associated estimates of uncertainty (e.g. confidence intervals)
☐	☒	For null hypothesis testing, the test statistic (e.g. *F*, *t*, *r*) with confidence intervals, effect sizes, degrees of freedom and *P* value noted<br>*Give P values as exact values whenever suitable.*
☒	☐	For Bayesian analysis, information on the choice of priors and Markov chain Monte Carlo settings
☒	☐	For hierarchical and complex designs, identification of the appropriate level for tests and full reporting of outcomes
☐	☒	Estimates of effect sizes (e.g. Cohen's *d*, Pearson's *r*), indicating how they were calculated

Our web collection on statistics for biologists contains articles on many of the points above.

Software and code

Policy information about availability of computer code

Data collection	BD FacsDiva version 6.1.3
Data analysis	FlowJo version 10.7.1.<br>Stata statistical software, version 15.1<br>Prism GraphPad version 9.1.1

For manuscripts utilizing custom algorithms or software that are central to the research but not yet described in published literature, software must be made available to editors and reviewers. We strongly encourage code deposition in a community repository (e.g. GitHub). See the Nature Portfolio guidelines for submitting code & software for further information.

Data

Policy information about availability of data

All manuscripts must include a data availability statement. This statement should provide the following information, where applicable:

- Accession codes, unique identifiers, or web links for publicly available datasets
- A description of any restrictions on data availability
- For clinical datasets or third party data, please ensure that the statement adheres to our policy

The data that support the findings of this study are available from the corresponding author upon reasonable request.

Human research participants

Policy information about studies involving human research participants and Sex and Gender in Research.

Reporting on sex and gender	We enrolled consecutive patients and did not specifically control for sex. Gender was not collected as part of the data ascertainment. In Cohort 1, 2, 3, and 4, the proportion of subjects with female sex were 25%, 35%, 38%, and 75% respectively. No specific analytical differences were found when analyzing outcomes based on sex.
Population characteristics	We prospectively recruited the following populations: (1) Infected organ transplant patients were recruited since the beginning of the SARS-CoV-2 pandemic. All patients that were prospectively recruited were asked to provide blood samples at convalescence. For analysis, this group was split into those that had non-Omicron BA.1 (Cohort 1) and Omicron BA.1 (Cohort 2) variants. (2) Vaccinated organ transplant patients that had received three doses of mRNA vaccine; (3) Healthcare workers that had received three doses of mRNA vaccination.
Recruitment	Transplant patients are followed closely by our hospital transplant center and were asked to report any positive COVID-19 diagnoses to our center. Therefore, patients that came to our attention were telephoned, consented, and asked to provide a convalescent blood sample. Healthcare workers were recruited by sending a mass email to healthcare workers. This was a prospective observational study and all healthcare workers provided written informed consent. The study cohort represents the demographics of healthcare workers at our institution and there is no apparent self-selection bias.
Ethics oversight	University Health Network Research Ethics Board

Note that full information on the approval of the study protocol must also be provided in the manuscript.

Field-specific reporting

Please select the one below that is the best fit for your research. If you are not sure, read the appropriate sections before making your selection.

☒ Life sciences ☐ Behavioural & social sciences ☐ Ecological, evolutionary & environmental sciences

For a reference copy of the document with all sections, see nature.com/documents/nr-reporting-summary-flat.pdf

Life sciences study design

All studies must disclose on these points even when the disclosure is negative.

Sample size	Sample size was determined using real world recruitment with convenience sampling. The infected cohorts (Cohort 1 and 2) were part of a large clinical data set of COVID positive transplant recipients. Within this dataset, some patients agreed to have blood collected and were included in this study.
Data exclusions	Data for T-cell analysis were excluded if the patient did not wish to provide additional blood for T-cell analysis.
Replication	Assays were not done in duplicate due to cost but all assays were validated and run with concurrent positive and negative controls.
Randomization	Not applicable.
Blinding	Not applicable. Laboratory personnel conducting the experiments were blinded to the clinical details of the subjects.

Reporting for specific materials, systems and methods

We require information from authors about some types of materials, experimental systems and methods used in many studies. Here, indicate whether each material, system or method listed is relevant to your study. If you are not sure if a list item applies to your research, read the appropriate section before selecting a response.

Materials & experimental systems

n/a	Involved in the study	
☐	☒	Antibodies
☐	☒	Eukaryotic cell lines
☒	☐	Palaeontology and archaeology
☒	☐	Animals and other organisms
☐	☒	Clinical data
☒	☐	Dual use research of concern

Methods

n/a	Involved in the study	
☒	☐	ChIP-seq
☐	☒	Flow cytometry
☒	☐	MRI-based neuroimaging

Antibodies

Antibodies used	Flow cytometry:<br>anti-human CD3-BV786 (BD Biosciences) clone SK7, cat no: 563799, dilution 1:80<br>anti-human CD4-Pacific Blue (BD Biosciences) clone RPA-T4, cat no: 558116, dilution: 1:40<br>anti-human CD8-APC-Cy7 (BD Biosciences) clone RPA-T8, cat no: 557760, dilution: 1:40<br>anti-human IFN-γ-FITC (BD Biosciences) clone B27, cat no: 554700, dilution: 1:40<br>anti-human IL-2-APC (BioLegend) clone MQ1-17H12, cat no: 500310, dilution: 1:40
Validation	All antibodies were obtained from commercial vendors and corresponding Data Sheets are available and provided by the manufacturers describing validation of specificity. All antibodies were separately titrated in-house to evaluate best concentrations for discrimination of negative and positive cells.

Eukaryotic cell lines

Policy information about cell lines and Sex and Gender in Research

Cell line source(s)	HEK293T cells were purchased originally from ATCC (ATCC #CRL-3216) and HEK293T-ACE2/TMPRSS2 cells were prepared in the lab according to a previously published protocol (Abe et al., 2020; JCI Insight)
Authentication	None of the cell lines used were authenticated.
Mycoplasma contamination	All cell lines used were tested for, and free of, Mycoplasma contamination.
Commonly misidentified lines<br>(See ICLAC register)	Not applicable

Clinical data

Policy information about clinical studies

All manuscripts should comply with the ICMJEguidelines for publication of clinical research and a completedCONSORT checklist must be included with all submissions.

Clinical trial registration	N/A - this was not a clinical trial
Study protocol	The full trial protocol can be made available by contacting the corresponding author.
Data collection	Patients were recruited from March 2020 to January 2022. Data was kept in a secure, password-protected excel file. Data was anonymized as not to include any patient identifiers. All data was collected prospectively.
Outcomes	Outcomes studied were a) anti-RBD antibodies, b) neutralization against D614G, Delta variant, BA.1, BA.2. c) T-cell frequencies for polyfunctional and monofunctional CD4+, CD8+ cells.

Flow Cytometry

Plots

Confirm that:

- [x] The axis labels state the marker and fluorochrome used (e.g. CD4-FITC).
- [x] The axis scales are clearly visible. Include numbers along axes only for bottom left plot of group (a 'group' is an analysis of identical markers).
- [x] All plots are contour plots with outliers or pseudocolor plots.
- [x] A numerical value for number of cells or percentage (with statistics) is provided.

Methodology

Sample preparation	PBMCs were isolated using conventional Ficoll-based separation protocols. After isolating the PBMCs from the peripheral blood, they were treated for 15 min in red cell lysis buffer according to the manufacturer's instruction (BD Pharmalyse). Cells were cryopreserved in 10% DMSO + 90% FBS (ThermoFisher Scientific). Cells were stored in a Mr. Frosty Freezing Container (ThermoFisher Scientific) for 24 hours at -80oC prior to storage in the vapor phase of liquid nitrogen. Samples were assessed in batches. Cells were gently thawed in a 37oC water bath and washed in complete RPMI (10% FBS, L-glu, pen/strep, HEPES) to remove residual DMSO. Cells were counted using a Vi-Cell instrument (Beckman Coulter) and seeded in 96-well plates at 1 million cells per well in complete RPMI. Cells were rested for 2 hours at 37oC prior to undergoing stimulation with/without peptides. Peptides were added at 5ug/mL, as determined by prior experiments and co-stimulatory antibodies were added (BD FastImmune anti-CD28/CD49d). An hour after stimulation of cells, a protein transport inhibitor was added to prevent cytokine release (ThermoFisher Scientific). Cells were incubated overnight (16 hours) and subsequently stained for viability using the Zombie Aqua viability dye (BioLegend) at 1:500 dilution. Cells were Fc blocked (BD Biosciences), and stained with a surface antibody cocktail consisting of human anti-CD3, -CD4 and -CD8 antibodies for 30 min on ice. Cells were then fixed in 4% paraformaldehyde (Fixation Buffer, BioLegend, Cat no: 420801) at room temperature for 30 min. Cells were incubated with an intracellular antibody cocktail targeting anti-human IFN-γ and IL-2 prepared in Intracellular Staining Permeabilization

	Wash Buffer (BioLegend) for 20 min at room temp. After staining, cells were washed thoroughly and resuspended in wash buffer. Tubes were wrapped in foil to protect from light, and kept at 4oC until acquisition of data. Data was acquired within 24 hours of staining.
Instrument	Data was analyzed on an LSR II VBGR instrument (BD Biosciences) or FACSymphony instrument (BD Biosciences) at the SickKids-UHN Flow Cytometry Core Facility.
Software	Acquisition was performed using BD FACSDiva. Analysis was performed using FlowJo, v10.7.1.
Cell population abundance	N/A. No sorting was performed.
Gating strategy	Gating Strategy: SSC-A vs FSC-A (lymphocytes) -> FSC-H vs FSC-A (single cells) -> count vs viability-Zombie Aqua (live cells; gated on Zombie Aqua-negative cells) -> count vs CD3-BV786 (CD3+ T-cells; gated on CD3+ cells) -> CD4-pacific blue vs CD8-APC-Cy7 (CD4+ T-cells are CD8-negative, and CD8+ T-cells were CD4-negative). Live CD4+ and CD8+ T-cells were then gated in terms of IFN-γ-FITC vs IL-2-APC to display cytokine production. To gate live/dead cells properly, control specimens were prepared where PBMCs were heat-killed by incubation at 65oC for 10 min and then mixed in a 1:1 ratio with live (non-heat killed cells). These cells were then stained for flow analysis. To determine the proper placement of cytokine gates for determining cut-offs, a combination of negative controls (unstimulated PBMCs) and positive controls (PBMCs stimulated with PMA/ionomycin for 16 hours, a known potent inducer of IFNg and IL2 production in T-cells) were used.

☒ Tick this box to confirm that a figure exemplifying the gating strategy is provided in the Supplementary Information.
